# Sec24D-Dependent Transport of Extracellular Matrix Proteins Is Required for Zebrafish Skeletal Morphogenesis

**DOI:** 10.1371/journal.pone.0010367

**Published:** 2010-04-28

**Authors:** Swapnalee Sarmah, Alejandro Barrallo-Gimeno, David B. Melville, Jacek Topczewski, Lilianna Solnica-Krezel, Ela W. Knapik

**Affiliations:** 1 Department of Medicine, Division of Genetic Medicine and Department of Cell and Developmental Biology, Vanderbilt University Medical Center, Nashville, Tennessee, United States of America; 2 Developmental Biology, Institute Biology I, University of Freiburg, Freiburg, Germany; 3 Department of Biological Sciences, Vanderbilt University, Nashville, Tennessee, United States of America; Max Planck Institute of Molecular Cell Biology and Genetics, Germany

## Abstract

Protein transport from endoplasmic reticulum (ER) to Golgi is primarily conducted by coated vesicular carriers such as COPII. Here, we describe zebrafish *bulldog* mutations that disrupt the function of the cargo adaptor Sec24D, an integral component of the COPII complex. We show that Sec24D is essential for secretion of cartilage matrix proteins, whereas the preceding development of craniofacial primordia and pre-chondrogenic condensations does not depend on this isoform. *Bulldog* chondrocytes fail to secrete type II collagen and matrilin to extracellular matrix (ECM), but membrane bound receptor β1-Integrin and Cadherins appear to leave ER in Sec24D-independent fashion. Consequently, Sec24D-deficient cells accumulate proteins in the distended ER, although a subset of ER compartments and Golgi complexes as visualized by electron microscopy and NBD C_6_-ceramide staining appear functional. Consistent with the backlog of proteins in the ER, chondrocytes activate the ER stress response machinery and significantly upregulate BiP transcription. Failure of ECM secretion hinders chondroblast intercalations thus resulting in small and malformed cartilages and severe craniofacial dysmorphology. This defect is specific to Sec24D mutants since knockdown of Sec24C, a close paralog of Sec24D, does not result in craniofacial cartilage dysmorphology. However, craniofacial development in double Sec24C/Sec24D-deficient animals is arrested earlier than in *bulldog/sec24d*, suggesting that Sec24C can compensate for loss of Sec24D at initial stages of chondrogenesis, but Sec24D is indispensable for chondrocyte maturation. Our study presents the first developmental perspective on Sec24D function and establishes Sec24D as a strong candidate for cartilage maintenance diseases and craniofacial birth defects.

## Introduction

Rapid availability of morphogens, receptors and extracellular matrix assures normal development and organ homeostasis. Secreted and membrane-bound proteins are synthesized in the ER and transported through the Golgi complex to their final destinations. The first step of ER-to-Golgi transport is mediated by the Coat Protein II complex, or COPII, which consists of a set of five core proteins, Sar1, Sec23, Sec24, Sec13 and Sec31, that are responsible for initiation of vesicle formation, cargo selection, polymerization and the release of loaded carriers from the ER membranes [1], [2]. We and others have shown that Sec23 proteins are required for development of the vertebrate skeleton, whereas other organs appeared less affected [3]–[6]. These findings suggest that secretion is highly regulated during development.

Vertebrate genomes contain four Sec24 genes in syntenic regions suggesting that the current gene families have evolved by tandem duplication events ([4], [6]–[8] and our unpublished data). This evolutionary expansion might have occurred concurrently with the acquisition of novel cargo proteins, e.g. extracellular matrix proteins that are not synthesized by unicellular organisms. *In vitro* studies and analyses in yeast and cultured mammalian cells uncovered that the Sec24 cargo adaptors bind preferentially to different ER export signals [9]–[12]. Interestingly, Hauri and colleagues [11] have shown, using model proteins in HeLa cells, that Sec24 isoforms exhibit selective binding preference for some export signals and a number of export signals are recognized in a redundant fashion by two or more of the Sec24 isoforms.

It appears that the repertoire of export signals recognized by close paralogs such as Sec24D and Sec24C shows a significant overlap. Furthermore, Sec24D does not seem to be essential for secretion in HeLa cells since other paralogs can substitute for its function. This work uncovered a new level of complexity in the regulation of the secretory pathway. However, how this complexity in target binding activity translates into physiological protein secretion during animal development has not been explored.

Here, we present data on the identification and cellular characterization of zebrafish *bulldog* mutations, which disrupts Sec24D function. *Bulldog* chondrocytes fail to secrete type II collagen and matrilin to extracellular matrix (ECM), but membrane bound receptor β1-Integrin and Cadherins appear to leave ER in Sec24D-independent fashion. We show that Sec24D is essential for extracellular matrix secretion in cartilage and that its deficiency results in failure of craniofacial morphogenesis to proceed past the initial stages of chondroblast development. Our work provides mechanistic insights into the role of Sec24D in the development of craniofacial structures.

## Results

### Mapping and Identification of the *bulldog* Mutations

**Figure 1:**
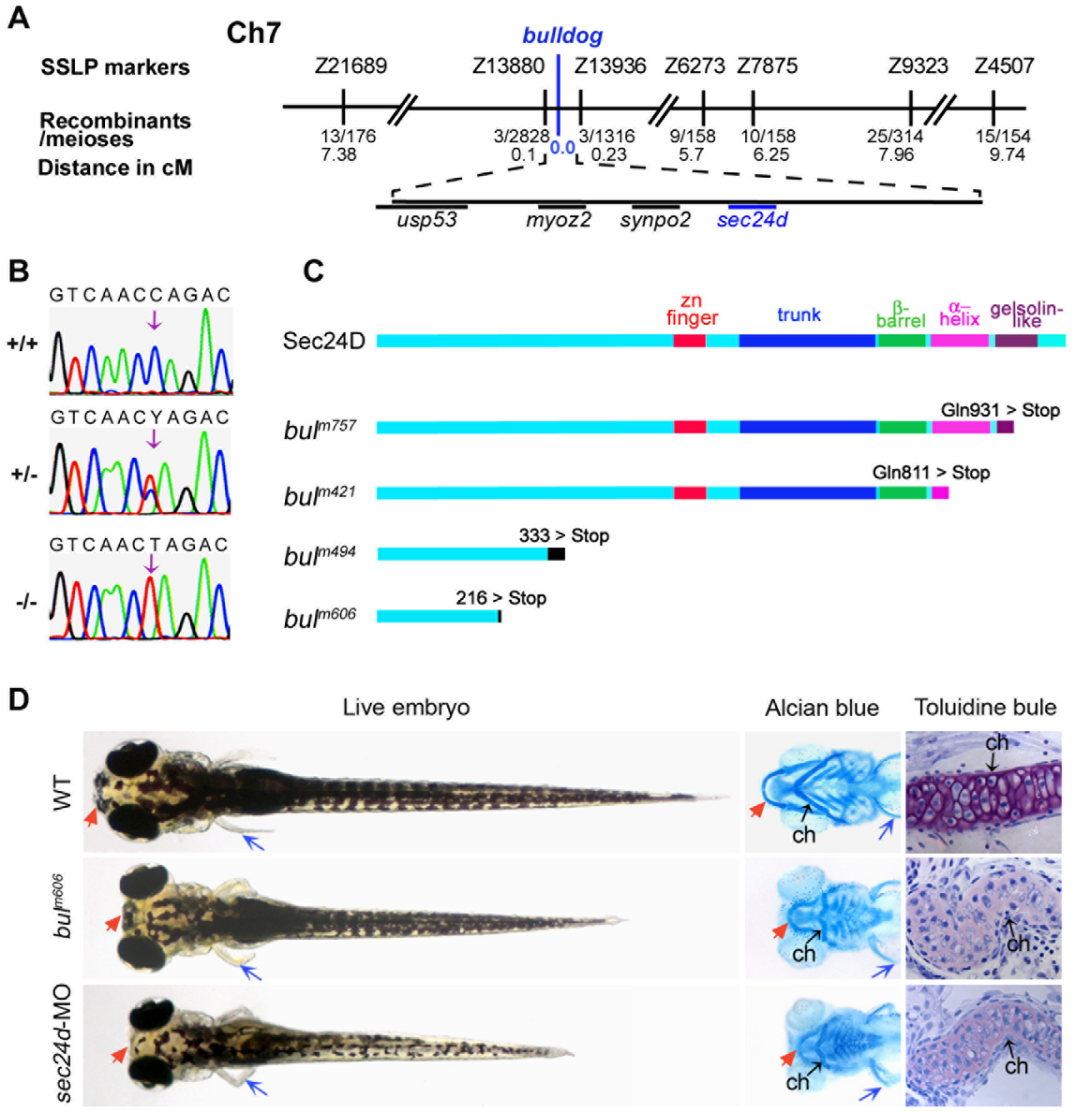
The *bulldog* mutations disrupt the *sec24d* gene and affect craniofacial development. (A) The *bulldog* mutations map to chromosome 7 between markers Z13880 and Z13936. The number of recombinants and the corresponding distances in centiMorgans (cM) are indicated below. The genes in the critical region are listed. (B) Electropherograms of wild-type +/+, heterozygous +/– and *bul^m421^* –/– genomic DNA shows the C→T transition (arrows) that results in an amber stop codon (TAG) in place of glutamine at position 811 (Q811X). (C) Schematic diagram of the Sec24D primary protein structure of wild-type and *bulldog* mutants. (D) *bul^m606^* and *sec24d* morphant embryos (3.5 ng *sec24d*-MO) have reduced head size (red arrows), shorter body length and pectoral fins (blue arrows). Alcian blue staining of the head skeleton in the right panels shows short Meckel's (red arrows), deformed ceratohyals (black arrows) and kinked pectoral fins (blue arrows) in *bul^m606^* and s*ec24d* morphant embryos compared to wild types. Toluidine blue stained coronal sections of craniofacial cartilage in *bul^m606^* and *sec24d* morphant embryos demonstrate very low amount of ECM material (purple staining) and abnormal shape and packing density of chondrocytes as compared to wild types, all at 4 dpf. Abbreviation: ch, ceratohyal.

*Bulldog* mutations were identified in a large-scale mutagenesis screen for embryonic lethal mutations disrupting craniofacial cartilage development [13]. To determine the molecular nature of the *bulldog* locus, we applied a positional cloning strategy using a ∼3,000 meioses F2 map cross and the microsatellite based genetic linkage map [14]. Using a genome-wide scan and 225 anchored microsatellite markers, we placed the four *bulldog* mutations on zebrafish chromosome 7 and subsequently restricted the critical interval to 0.33 cM between markers Z13880 and Z13936 (Figure 1A). We found that Z13880 is in an intron of the zebrafish ortholog of the human *USP53* gene that is located near the *SEC24D* locus in both the human and *Fugu* genomes. We confirmed by radiation hybrid mapping that the two genes are also in syntenic positions in the zebrafish genome (data not shown). Based on the *bulldog* phenotypic data and the *sec24d* position, at a zero recombination distance from the mutation, we sequenced *sec24d* as a suitable candidate gene.

The putative zebrafish Sec24D protein contains five functional domains (from N- to C-terminus) – zinc finger, trunk, β-sandwich, α-helix and gelsolin-like – that are highly conserved across different species [7], [8] (Figure S1). We determined that the lesions in the four *bulldog* alleles are predicted to cause increasingly longer truncations of the Sec24D protein removing successive functional domains (Figure 1B,C). We identified a C→T transition in *bul^m757^* genomic DNA that is predicted to introduce an amber stop codon (TAG) in place of glutamine at position 931 (Q931X); a C→T transition in *bul^m421^* predicted to result in a Q811X change; an exon 7 deletion in *bul^m494^* leading to a frame shift and a stop codon at amino acid 333; and a C→A transversion in *bul^m606^* that abolishes the 3′ splice junction of intron 4 leading to a frame shift and a premature stop codon at amino acid 216.

### Skeletal Dysmorphology of the *bulldog* Mutants

The phenotype of the *bulldog* mutant embryos features a shorter body length, kinked pectoral fins and a shorter and misshapen head skeleton (Figure 1D). Whole mount preparations of head skeletons stained with Alcian blue showed that all cartilage elements are present, indicating normal progress of early patterning steps of skeletogenesis. However, the elements appear to be shorter, misshapen and not correctly aligned in respect to each other and the body axes, suggesting defects in later developmental steps of cartilage maturation. This is consistent with the fact that the *bulldog* phenotype can be first distinguished at approximately 60 hpf (hours post fertilization). Histological sections stained with toluidine blue (dye marking cartilage proteoglycans) showed reduced staining of extracellular matrix and pronounced cartilage dysmorphology with accumulation of purple stained matrix within chondrocytes (Figure 1D).

To confirm that *sec24d* is the mutated gene in *bulldog*, we depleted Sec24D in wild-type embryos using antisense morpholino oligonucleotides (MO). We designed MO against the *sec24d* 5′UTR sequence in order to interfere with translation of both maternal and zygotic transcripts. *sec24d*-MO injections resulted in a set of defects phenocopying the *bulldog* mutants (Figure 1D). However, morphants were slightly smaller than *bulldog* embryos, suggesting that maternal transcripts might have a minor contribution during early development ([15]; see below). Overall, the genetic mapping data, the indistinguishable phenotypes of the four *bulldog* alleles, and the similar perturbations produced by the antisense interference led us to conclude that *bulldog* disrupts Sec24D function.

### Expression of Sec24D During Embryonic Development

**Figure 2:**
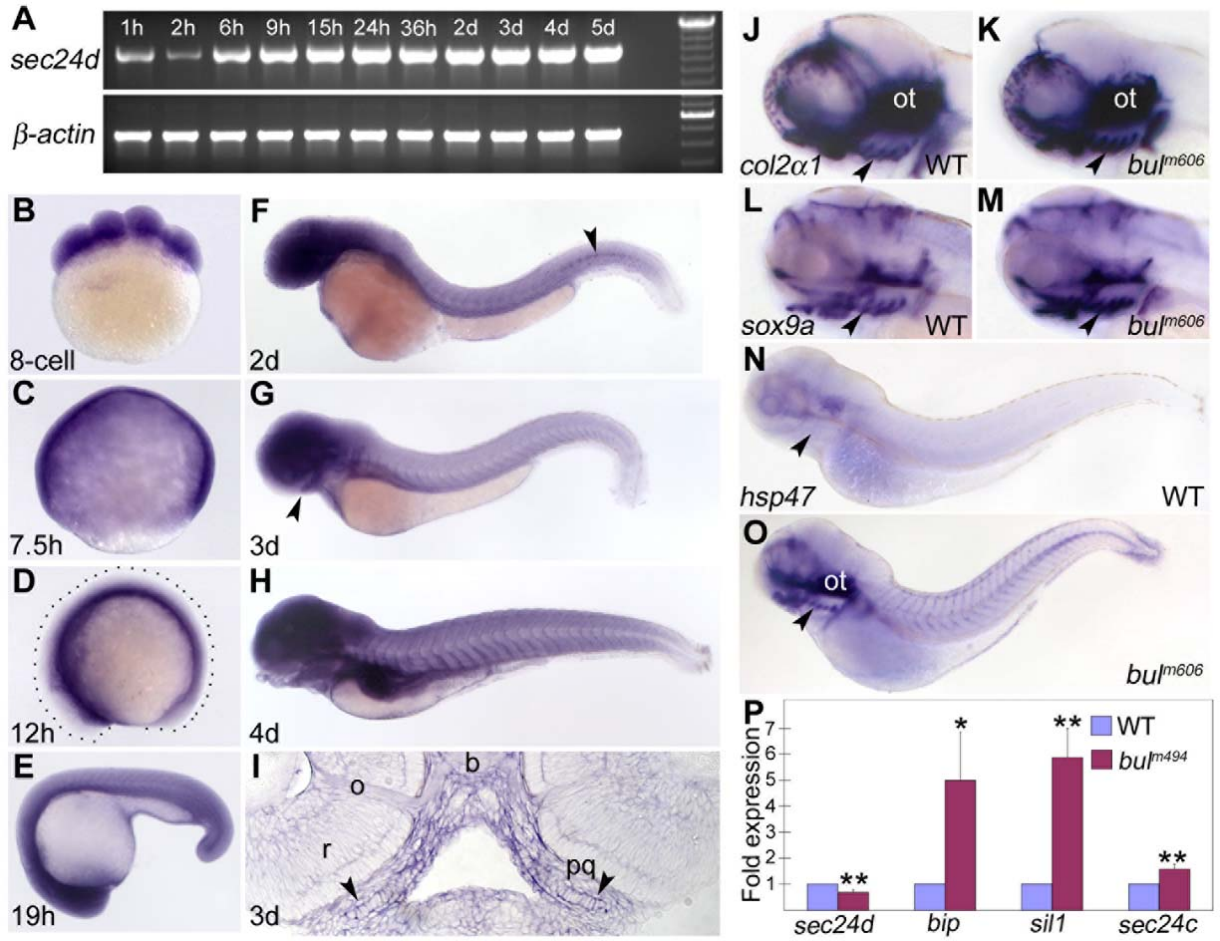
*sec24d* mRNA expression during embryogenesis and molecular analysis of the *bulldog* mutation. (A) RT-PCR assay shows maternally deposited *sec24d* transcripts at 1 and 2 hpf and increasing levels thereafter. β-actin served as control. (B–H) Expression patterns of *sec24d* during zebrafish development using whole mount *in situ* hybridization staining of wild-type embryos. The cross section through the palatoquadrate (pq, arrowheads) at 3 dpf (I) shows robust riboprobe staining (h: hours, d: days). (J–O) Expression patterns of *col2*α*1* (J,K) and *sox9a* (L,M) at 3 dpf. The expression is slightly upregulated in the posterior pharyngeal cartilages of *bul^m606^* embryos. The Collagen-specific chaperone *hsp47* (N,O) transcript is elevated in craniofacial skeleton (arrowheads), fins and the notochord. Arrowheads point to the jaw region in panels (G) through (O). (P) Relative *sec24d sec24c, bip* and *sil1* mRNA levels were assessed by quantitative real-time PCR and adjusted against β-actin using total RNA samples from 4 dpf embryos. In *bul^m494^,* mRNA levels for *bip* and *sil1* are induced 5-fold, whereas *sec24d* mRNA levels are reduced 0.7-fold. *, *p*<0.02; **, *p*<0.005. Abbreviation: b, brain, o, optic nerve, ot, otic capsule, r, retina. Anterior (D–O) is to the left.

To examine whether the spatio-temporal expression pattern of *sec24d* correlates with the developmental defects in *bulldog* mutants, we analyzed RNA samples from consecutive stages by RT-PCR and embryos by *in situ* hybridization. We found *sec24d* expression at 1 hpf, which subsequently decreases prior to the onset of zygotic transcription at 3 hpf confirming maternal deposition of *sec24d* transcripts (Figure 2A,B). *sec24d* levels were uniformly distributed during the cleavage (Figure 2B) and gastrula stages (Figure 2C). Later in development, *sec24d* expression was detected throughout the embryo including the head (brain, eye, cartilage), notochord, intersomitic boundaries, and gut (Figure 2D–I). At day 3, expression was prominent in the craniofacial cartilage, consistent with the timing of the deficits in the mutant and morpholino phenotypes.

### *bulldog* Upregulates Collagen Expression and Triggers ER Stress Response

To uncover how the *bulldog* mutations in the *sec24d* gene affect the development of chondrocytes, we investigated the expression of *collagen2α1* (*col2α1*), the primary cartilage matrix protein, and *sox9a,* a transcription factor known to direct *col2α1* expression in developing chondrocytes [16], [17]. *In situ* hybridization with riboprobes recognizing the respective transcripts showed that, in *bulldog*, expression of *sox9a* and *col2α1* was slightly elevated in the posterior pharyngeal arches (Figure 2J–M). Furthermore, Hsp47, a chaperone essential for procollagen processing in the ER [18], was expressed at significantly higher levels in *bulldog* mutants relative to wild types (Figure 2N,O).

The gene expression results suggested that *sec24d* inactivation led to stimulation of the collagen biosynthesis pathway, which could trigger ER-stress response [19]. To test this hypothesis, we analyzed expression of *bip* and *sil1* (a co-chaperone for BiP), two components of the ER quality control pathway, by quantitative PCR at consecutive stages from 3 to 6 dpf (Figure 2P and data not shown). We found that both genes were significantly upregulated in *bulldog* mutants compared to the wild-type baseline, with *bip* and *sil1* levels being increased approximately 5-fold. Furthermore, *sec24d* transcript levels were slightly decreased, likely a result of nonsense-mediated decay. However, *sec24c* expression was slightly increased in *bulldog* mutants, implying activation of possible compensatory mechanisms. Taken together, the *bulldog* mutations in Sec24D result in increased synthesis and processing of type II collagen and upregulation of the ER stress response machinery, suggesting a deficit in the secretory pathway during craniofacial morphogenesis.

### Morphology of ER Membranes in Sec24D-deficient Chondrocytes

**Figure 3:**
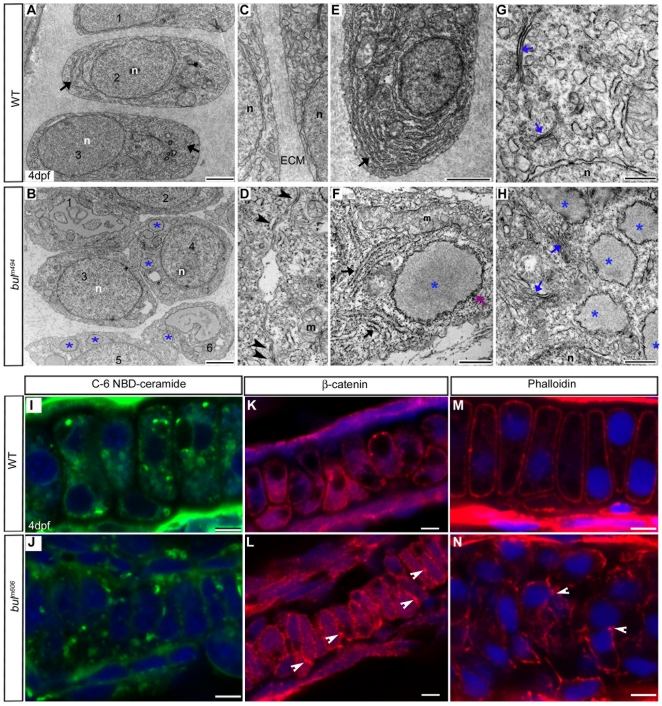
Sec24D-deficient cartilage contains sparse ECM and chondrocytes with distended ER. (A–H) Electron micrographs show mature wild-type chondrocytes (cells 1–3 in A) are separated by ECM and contain dense ER membranes (A, E, arrows). In *bul^m494^* mutants (B,F,H), cartilage matrix is sparse and large amounts of electron-dense material accumulate in the rough ER (asterisks, cells marked 1–6 in B). Wild-type chondrocytes (C) are devoid of cell-cell contacts, whereas the majority of *bul^m494^* mutant cells (D) contain adherens junctions (arrowheads). *bul^m494^* chondrocytes (F) contain both normal ER cisternae (arrows) and swollen, ribosome studded, ER (asterisks). *sec24d*-deficient cells (H) contain smaller and disorganized Golgi complexes (blue arrows) compared to wild-types (G). (I,J) Single-pass confocal images of C_6_-NBD ceramide stained chondrocytes of wild-type (I) and *sec24d/bul^m606^* mutant (J) embryos at 4 dpf. (K,L) Adherens junctions are marked by β-catenin (*red*) in *bulldog* Meckel's cartilage (arrowheads), but are mostly absent in wild types (K). (M,N) Phalloidin (*red*) shows regular cortical distribution of polymerized actin in wild types (M), but uneven cellular distribution in *bul^m606^* cartilage (arrowheads) (N). Nuclei are stained with TOPRO-3 (*blue*). Abbreviations: m, mitochondria; n, nucleus; ECM, extracellular matrix. Scale bars: 2 µM (A,B,E); 500 nm (F–H); 5 µM (I–N).

To understand how Sec24D deficiency affects chondrocyte morphology and function, we analyzed transmission electron micrographs (TEM) of *bulldog* embryos at 2.5, 4 and 6 dpf (Figure 3A–H; and data not shown). Although chondrocytes in wild-type embryos were elongated, neatly stacked, and separated from each other by dense ECM (Figure 3A,C,E), *sec24d*-deficient cells were small, round, in shape and attached to each other. The cells contained distended ER filled with electron dense material and were surrounded by in sparse ECM (Figure 3B,D,F). Interestingly, *sec24d*-deficient chondrocytes contained both normal and distended ribosome studded ER membranes (Figure 3F) and stacks of juxtanuclear cisternae associated with vesicles, typical of Golgi complexes (Figure 3G,H). Although the cisternae in *bulldog* were smaller in size and not as densely packed as the wild-type Golgi complexes, they appeared to be functional based on normal localization of C_6_-NBD ceramide (Figure 3I,J).

TEM analysis revealed cell-cell junctions at 4 dpf in *bulldog* mutant embryos that are typical for condensing chondroprogenitors at earlier developmental stages [16], [17] (Figure 3C,D). We investigated whether the primary components of the adherens junctions N-cadherin, and β-catenin, are present at this time point and found that Sec24D-deficient cells maintain strong, consistent membrane localization of cadherins and β-catenin (Figure 3K,L and Figure S2). On the contrary, wild-type cartilages, which contain a mixture of cells in progressively more differentiated states, some of which already lost cell-cell contacts, expressed significantly lower levels of β-catenin and cadherins.

The transmembrane cadherin and associated cytoplasmic β-catenin complexes are anchored by the actin cytoskeleton on the inside of the cell membrane and are needed to maintain cell shape [20]. Phalloidin labeling of actin microfilaments showed dense, cortical microfilaments just inside the plasma membrane of wild-type chondrocytes. In Sec24D-deficient cells, however, the filaments appeared disorganized, and cells exhibited random shapes and maintained an uneven distribution of actin at cell-cell junctions (Figure 3M,N). Taken together, our results suggest that *bulldog* chondroblast differentiation fails to progress to the stage when they are separated by ECM, resulting in an earlier morphological phenotype in mutant embryos.

### Quantification of Cell Shape

**Figure 4:**
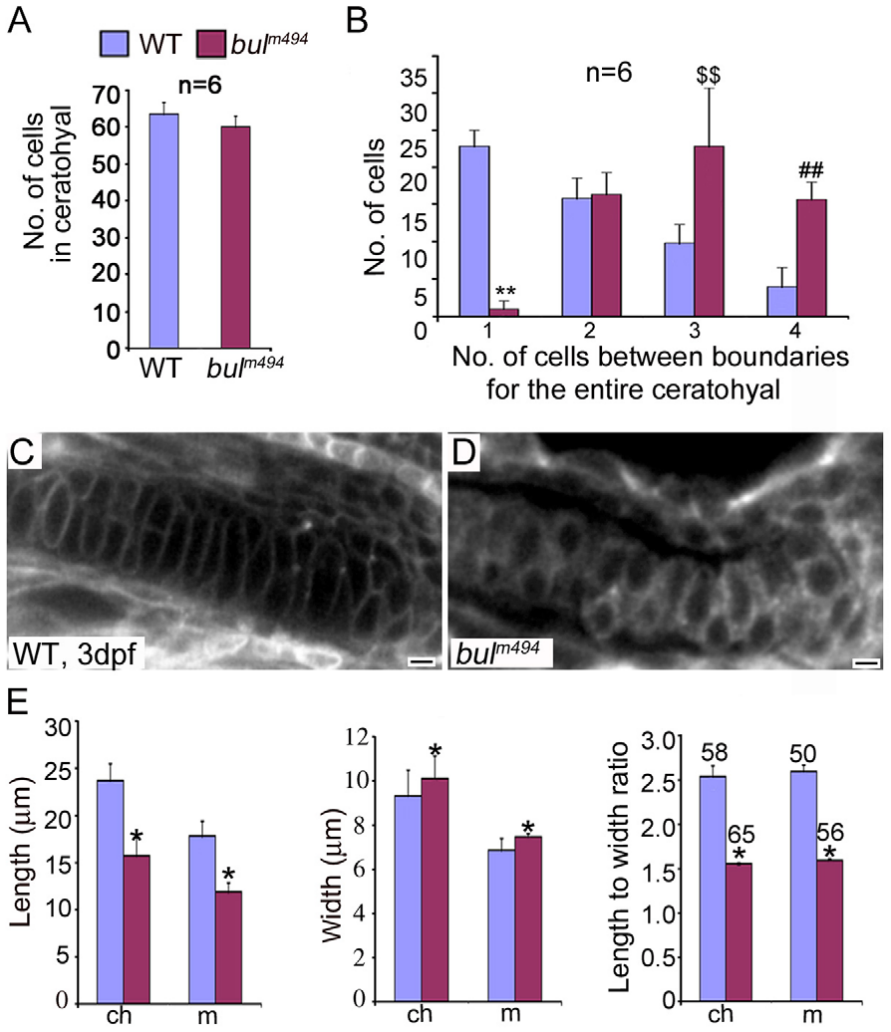
Analysis of chondrocyte shape and numbers in *bulldog* mutants. (A) The number of cells in the ceratohyals at 5 dpf is not significantly different between wild-type and *bulldog* embryos (counted in single optical plane of Alcian blue stained preparations, six different animals each). (B) In contrast, the number of cells spanning the entire width of the ceratohyal at 5 dpf is notably higher in *bulldog*. (C,D) Single-pass confocal images of the Meckel's cartilage in live embryos marked with membrane tethered GFP tracer. *Bulldog* mutants (D) show multiple stacked chondrocytes as compared to a single spanning cell in wild-types (C). (E) The average chondrocyte width is comparable between wild-type and *bulldog,* whereas the length and the length-to-width ratio are significantly lower in the mutants. Cellular dimensions were counted in Meckel's (m) and ceratohyal (ch) cartilages in three different live embryos at 3 dpf. The number of cells used for measurements is indicated in the right graph (E). * denotes *p*<0.0001; **, *p*<0.0001; $$, *p*<0.003; ##, *p*<0.0001.

Transition from condensed chondroprogenitors to differentiating cartilage elements is marked by changes in cell shape and their arrangement along three axes. To quantify the extent of cartilage dysmorphology, we first counted the number of cells in individual cartilage elements in Alcian blue preparations, but we found no significant differences between wild-type and *bulldog* embryos (Figure 4A). Because the principal mechanism of cartilage morphogenesis is by intercalation of condensed chondroprogenitors [21], we assessed the progress of intercalations by counting the number of cells spanning the width of the ceratohyal (Figure 4B). We found that this process is essentially stalled in Sec24D-deficient cartilage. Unlike the cellular organization in wild-type ceratohyals, where one or two chondrocytes spanned the width of the cartilage, in *bulldog,* mostly three and four cells were aligned perpendicular to the long axis of the cartilage. This arrangement, which largely resembls clusters of condensed chondroprogenitors, persisted in older larvae, further supporting the notion that the Sec24D deficiency arrested chondrocyte maturation.

To assess the shape and growth of chondrocytes, we measured cell length and width and calculated the length-to-width ratio in live embryos labeled with membrane tethered GFP (Figure 4C,D). The results show that the length-to-width ratio is approximately 40% lower in *bulldog* chondrocytes as compared to wild-type cells (Figure 4E). Moreover, the *bulldog* cells are shorter and wider than wild-type chondrocytes. It is likely that the failure of chondrocytes to intercalate stems from insufficient deposition of cartilage matrix to pericellular space, leading to faulty cell-matrix interactions required to remodel adherens junctions and alter cell shape [16], [17]. In addition, it is also likely that defective matrix composition compromises the function of associated growth factors (e.g., Wnts) that are needed for proper chondroblast maturation [22]–[24].

### Sec24D is not Essential for Mesenchymal Condensations

The results described above suggest that Sec24D deficiency affects general protein transport during the late stages of cartilage development. Alternatively, *bulldog* might selectively disrupt transport of specific types of cargo proteins, such as matrix proteins, but not transmembrane cell adhesion molecules throughout craniofacial morphogenesis. To test these hypotheses, we examined the cellular localization of selected proteins at different time points corresponding to the prior condensation and subsequent maturation stages.

**Figure 5:**
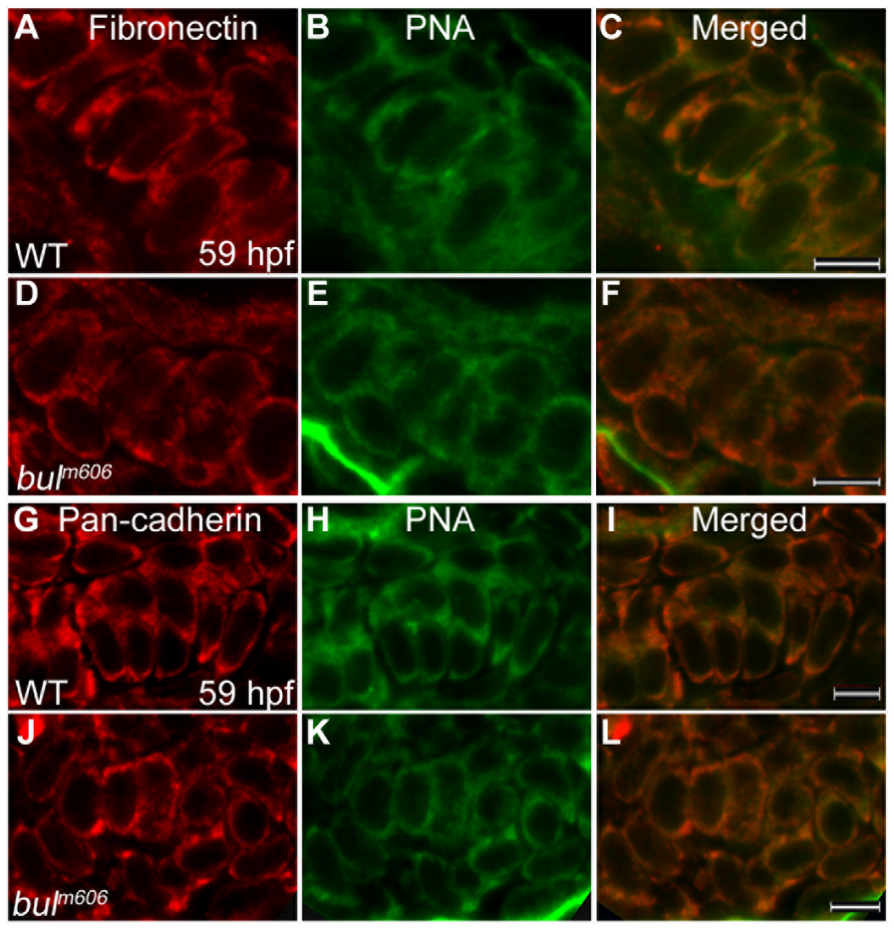
Analysis of mesenchymal condensations in cartilage primordia of *bulldog* mutants. (A–F) Mesenchymal condensations, stained with Peanut Agglutinin (PNA, *green*) show similar distribution of Fibronectin (*red*) in wild-type (A–C) and *bulldog* hyosymplectic cartilages (D–F). (G–L) Cadherin expression in chondrocytes (marked by a Pan-cadherin antibody in *red*) is indistinguishable between wild-type and *bulldog* ceratohyals as shown by PNA staining. The right panels represent merged images of the left and middle panels. Scale bars are 5 µM.

To visualize mesenchymal condensations, we used peanut agglutinin (PNA), a lectin that preferentially binds to the cell surface of condensing cells [25], [26]. Concomitantly, we analyzed the cellular distribution of Fibronectin (extracellular matrix protein) and Cadherin (transmembrane cell adhesion molecule) at 59 hpf. Both these proteins are highly expressed during mesenchymal condensations. The 59 hpf was chosen because this is the earliest time point when we can distinguish *bulldog* from wild-type phenotype by gross morphology. Condensation centers for different cartilage elements form over a period of several hours, so the jaw preparations present a range of stages at any given time (Figure S3.) We found that the overall cellular morphology and the distribution of the two proteins were indistinguishable between wild-type and *bulldog* fish (Figure 5). Thus, it appears that Sec24D activity is not essential for trafficking of all extracellular matrix or transmembrane proteins at the condensation step of cartilage morphogenesis.

### Sec24D Activity is Required for Effective Transport of Cartilage Matrix Proteins

**Figure 6:**
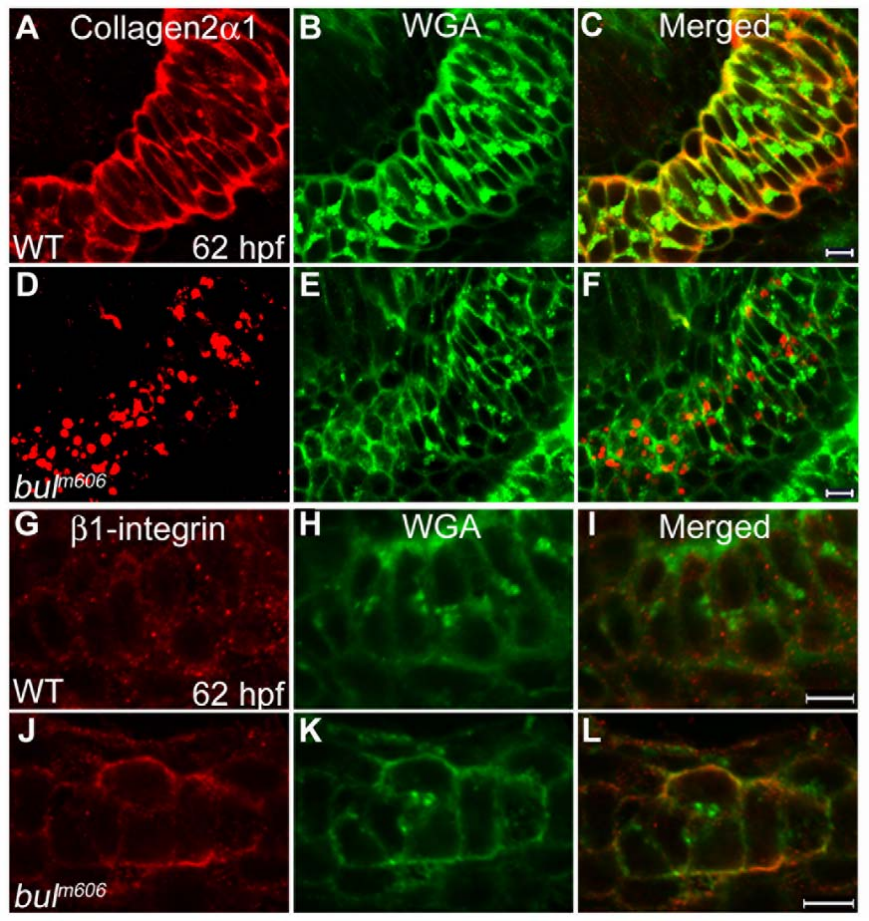
Sec24D-deficient chondrocytes do not transport ECM proteins. (A–F) Collagen2α1 (*red*) and Wheat Germ Agglutinin (WGA, *green*) signals are concentrated at juxtanuclear compartments and in the extracellular space of wild-type chondrocytes (A–C). In *bulldog*, the signals are primarily localized in cytoplasmic vesicular compartments, although weak WGA signal is also detectable at the plasma membrane and the extracellular space (D–F). (G–L) Single pass confocal images of wild-type and *bul^m606^* Meckel's cartilages labeled with the β1-integrin (*red*) recognizing antibody and counterstained with WGA (*green*). Merged channels show co-localization of the two labels in the cell boundaries in both wild-type and *bulldog* chondrocytes. The right panels represent merged images of the left and middle panels. Scale bars are 5 µM.

To assess protein transport during chondrocyte maturation, we examined the distribution of Collagen2α1 at 59, 62 hpf, and 3, 4, 5 dpf embryos [27]. To label individual skeletal elements, we used wheat germ agglutinin (WGA) [6]. In the wild-type chondrocytes, Collagen2α1 and WGA-labeled glycoproteins were primarily localized to the extracellular space and small juxtanuclear compartments (Figure 6A–C). On the contrary, in *bulldog* mutants, Collagen2α1 was not detectable in the extracellular space but accumulated in numerous, large vesicular structures within chondrocytes (Figure 6D–F; Figure S3 and not shown). Notably, most of the collagen-positive vacuolar structures did not accumulate WGA-positive glycoproteins at 59 and 62 hpf. However, at consecutive days of cartilage maturation, the two staining patterns began to co-localize, suggesting that some of the WGA-positive glycoproteins became backlogged (Figure S3). Similar accumulation within the cells was observed for the structural cartilage matrix protein Matrilin [28] (Figure S3).

To test whether the transmembrane receptors interacting with type II collagen are present at the cell surface, we examined the cellular localization of β1-integrin [29]. Confocal images revealed that the cellular distribution of β1-integrin is largely similar between wild types and *bulldog* mutants, without evident accumulation in the pre-Golgi compartment of *bulldog* (Figure 6G–L). However, in *sec24d* mutants, the β1-integrin puncta are less prominent than in wild types. Cytoplasmic localization of β1-integrin likely results from internalization of the receptor from the cell surface by the endocytic pathway, a process known to maintain cell-matrix homeostasis and matrix remodeling [30].

In summary, our results indicate that proteins involved in mesenchymal condensations are trafficked along the secretory pathway in Sec24D-independent fashion regardless of their type or final destination. This is further confirmed by the normal progression of mesenchymal condensations in *bulldog* mutants. In contrast, during the subsequent cartilage maturation stage, structural matrix proteins, such as type II collagen and Matrilin, require Sec24D for efficient ER export, whereas membrane receptors such as β1-integrin do not. These results suggest that Sec24D is essential for secretion of matrix proteins during the final stages of cartilage differentiation.

### Sec24C Compensates for Sec24D Loss During Early Craniofacial Development

**Figure 7:**
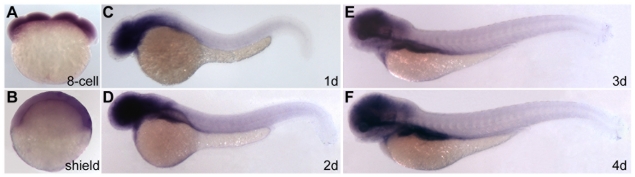
Sec24C expression during zebrafish development. (A,B) *sec24c* is ubiquitously expressed at cleavage (8-cell, A) and gastrulation (shield, B) stages. (C–F) *Sec24c* expression at consecutive embryonic days is concentrated in the head and gut regions. Expression in the head overlaps with developing craniofacial structures.

Vertebrate genomes contain four Sec24 genes, which can be aligned in pairs, Sec24D/Sec24C and Sec24B/Sec24A. These pairs retain high sequence similarity in addition to preferential binding of similar ER export signals as shown in yeast and in cultured cells *in vitro* using model proteins [8]–[11]. Because the zebrafish genome does not appear to harbor duplicated Sec24D genes (Zebrafish Genome Sequencing Project, NCBI), we asked whether Sec24C is also important for craniofacial morphogenesis and chondrocytes maturation. To test this possibility, we cloned zebrafish *sec24c* cDNA and synthesized riboprobes to assess its expression pattern (Figure 7). We found that *sec24c* was maternally deposited and broadly expressed throughout the head and developing jaw region during the first 48 hpf and remained present during the following two days, albeit at lower levels compared to earlier time points.

**Figure 8:**
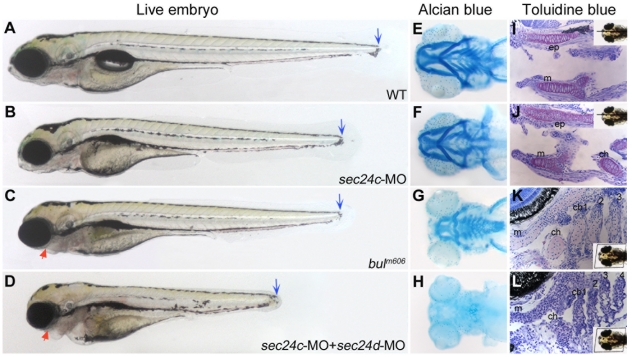
Knockdown of *sec24c,* and combined loss of *sec24d* and *sec24c* phenotypes. Analysis of wild types (A, E, I), *sec24c*-UTR-MO (B,F,J), *bulldog* (C, G, K) and double *sec24c*-UTR-MO+ *sec24d*-UTR-MO (D, H, L) injected embryos. (A–D) Images of live wild types (A), *sec24c*-UTR-MO, (2.0 ng injected, B), *bulldog* (C) and *sec24c*-UTR-MO+ *sec24d*-UTR-MO (2.0 ng each, D). The *sec24c* morphants have reduced body length (*blue arrows*), but the head appears normal. Double morphants for *sec24d+sec24c* are significantly shorter than *bulldog* larvae, almost completely lack fin-folds and have reduced jaw region (*red arrow*) and pronounced heart edema (D). (E–H) Alcian blue preparations of the head skeleton. The Alcian blue staining confirms normal craniofacial cartilages in *sec24c* morphants. (I–L) Histological sections counter-stained with toluidine blue at 4 dpf. Insets indicate the plane of the sections. (I,J) Histological, sagittal plastic sections stained with toluidine blue show normal jaw opening and surrounding cartilages in wild types (I) and *sec24c* morphants (J). Purple staining patterns for glycosaminoglycans of the ECM are also comparable. In contrast, the pharyngeal skeleton of *sec24d+sec24c* double mutants fails to stain with Alcian blue (H). Histological sections reveal patterned arches that are largely devoid of metachromatically stained cells (L). Abbreviations: cb, ceratobranchial; ch, ceratohyal; ep, ethmoid plate, m, Meckel's cartilage.

To test the role of Sec24C in craniofacial development, we knocked down Sec24C activity using a morpholino-based strategy (Figure S4). Although *sec24c* morphants were shorter than wild types, their head skeleton appeared normal in live embryos and in Alcian blue stained preparations (Figure 8). Toluidine blue counter stained histological sections revealed well-patterned cartilages with normal distribution of cartilage matrix. Thus, it appears that Sec24C is not essential for craniofacial cartilage morphogenesis.

**Figure 9:**
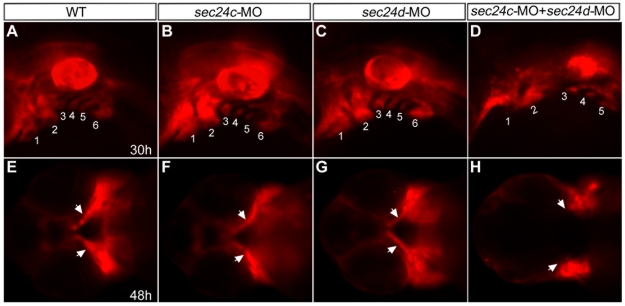
Migration of craniofacial primordia in double Sec24C and Sec24D morants. (A–H) Images of transgenic sox10:mRFP embryos at 30 hpf (lateral view A–D) and 48 hpf (ventral view E–H) after injection with *sec24c*-UTR-MO, (2.0 ng), *sec24d*-UTR-MO (3.5 ng), and *sec24c*-UTR-MO+ *sec24d*-UTR-MO. Neural crest streams are numbered (1, 2, 3, 4, 5, 6 in A–D). Arrows point to migrating craniofacial primordia (E–H).

Single knockdown of endogenous SEC24C or SEC24D failed to produce detectable transport phenotypes in HeLa cells [11]. However, the combined depletion of the two isoforms was shown to affect secretion of model proteins containing V-mediated transport motifs. To test whether the Sec24C and Sec24D functionally compensate for each other during zebrafish development, we investigated the compound effects of Sec24C and Sec24D inactivation. Injection of morpholinos targeting both isoforms resulted in well-patterned embryos at 24 hpf but progressively shorter embryos at later stages, with defects in the jaw region, ear capsule, pectoral fin skeleton and fin-fold, all structures known for high matrix content (Figure 8D). In Alcian blue preparation we found that the cartilaginous skeleton was almost absent. However, histological sections showed that structured pharyngeal arches were present, but they contained very few chondrocyte-like cells (Figure 8H,L). These results prompted us to investigate which step of craniofacial morphogenesis depends on combined activity of Sec24D and Sec24C. To this end, we used the transgenic zebrafish line *Tg(sox10(7.2):mrfp)*, which marks migratory neural crest cells [31] and injected *sec24d*, *sec24c*, and combined *sec24d* plus *sec24c* morpholinos (Figure 9 and not shown). We inspected 6 consecutive stages of facial primordia formation in live embryos (17 h, 26 h, 30 h, 48 h, 3 d and 4 d) and found that each of the individual morphants developed normally and was indistinguishable from un-injected controls at 48 hpf. As expected, Sec24C morphants retained wild type appearance throughout the observation period, whereas Sec24D morphants showed the first defects shortly after the 48 hpf time mark. Double morphants showed normal patterning of migratory neural crest up to 26 hpf and only slightly delayed migration at 30 hpf. However, there was a clearly visible failure to extend the first and the second arch primordia at 48 hpf. Our results indicate that Sec24C and Sec24D functionally compensate for each other during the early, mesenchymal condensation stages of craniofacial morphogenesis, whereas, Sec24D is solely required for effective cartilage matrix deposition.

## Discussion

Protein transport from the site of synthesis to the extracellular space or plasma membrane is a fundamental cellular process. The COPII components are essential for survival in yeast, and COPII vesicles are a universal feature in animal and plant cells, suggesting an evolutionarily conserved role in intracellular protein trafficking.

Recent phenotype-driven forward genetic screens in animal models and identification of genes causing human syndromes revealed that COPII mutations give rise to tissue and cell-specific deficits [3], [5], [6], [32], [33]. These findings have suggested that the various COPII components have specialized functions in higher organisms.

### The *bulldog* Mutations Disrupt Sec24D Function

Our results show that inactivation of the COPII component Sec24D leads to a specific defect in zebrafish chondrocyte development. Secretion and deposition of extracellular matrix proteins such as Collagen II and Matrilin is practically absent, leading to misshapen cartilage elements with embedded immature chondrocytes in disarray. Protein transport appears to proceed normally during the mesenchymal condensation stage of cartilage morphogenesis. Also, transfer of plasma membrane receptors and cell adhesion molecules seems to be undisturbed throughout cartilage development. However, Sec24D is indispensable for ECM deposition during chondrocytes differentiation.

It is conceivable that the restricted deficits reflect partial loss of function or significant contribution of maternally deposited proteins. However, this might not be the case for the following reasons. First, we have analyzed four independent ENU-induced *bulldog* mutations (*bul^m757^, bul^m606^, bul^m494^, bul^m421^*). All mutations in this allelic series result in a predicted STOP codon and progressively longer truncations of the 1030 amino acids long protein. Specifically, *bul^m757^* and *bul^m421^* are point mutations that create STOP codons resulting in shortened peptides missing the last 99 and 219 amino acids, respectively; whereas *bul^m606^* and *bul^m494^* are splice site frame shift mutations resulting in predicted premature stop codons at the beginning of the protein at amino acids 216 and 333, respectively. All mutants result in practically identical phenotypes, as assayed by histology, immunohistochemistry and *in situ* hybridization, suggesting that the C-terminus of Sec24D, the common deletion in all mutants, is critical for protein folding and dimerization. Consistent with this idea, crystal structure analysis of the yeast Sec24 protein has shown that the last 200 residues contribute to the gelsolin-like and helical domains, which reside at the center of the tertiary structure of the protein and are surrounded by the remaining functional domains. Thus, it is likely that the mutated proteins fail to fold and remain inactive [34]. These findings strongly support the notion that *bulldog* mutants represent genetic null alleles. Furthermore, the similar phenotypes of *bulldog* embryos and *sec24d-*morphants generated by translation blocking antisense oligonucleotides corroborate this assumption.

A concern in the analysis of gene mutations in zebrafish is the possibility of duplicated genes, but we did not found duplicated copies of the *sec24d* gene in zebrafish (Zv8, Sanger Center). In addition, it is also possible that maternal deposition of wild type transcripts and proteins in the egg could rescue early embryonic phenotypes. Activation of zygotic transcription in zebrafish occurs concurrently with degradation of maternal mRNAs during the midblastula transition at 3 hpf [35], however, the mechanisms controlling these processes, the amounts of maternal dowry and the turnover times are largely unknown and appear to be gene specific. We found that *sec24d* transcripts are maternally deposited. To gauge the effects of maternal transcripts on early development, we injected translation-blocking morpholinos into sensitized *bulldog* background and found similar phenotypes to the *bulldog* mutants. This result suggests that maternal Sec24D plays a minor role during early developmental stages.

### Sec24D has a Critical Role in ECM Transport of Differentiating Chondrocytes

The *bulldog* mutations are the first animal model to shed light on the function of Sec24D during embryonic development. Several lines of evidence support the hypothesis that Sec24D acts in a cell-, differentiation stage-, and cargo-specific manner. First, although *sec24d* is ubiquitously expressed during gastrulation, and broadly present at later stages of development, mutant embryos are well-shaped and the phenotype is most prominent in the craniofacial and pectoral fin skeleton. Second, craniofacial development proceeds relatively normally during the early stages with sufficient transport of cadherins and β-catenin to the plasma membrane and Fibronectin to the extracellular space. In contrast cartilage matrix secretion, including Collagen2α1 and Matrilin, during the onset of chondrocyte maturation depends on Sec24D activity. And third, certain protein types such as β1-integrin are transported to the plasma membrane throughout cartilage development, suggesting that other adaptors are sufficient for β1-integrin trafficking.

It is conceivable that Sec24D function is critical in cells with high cargo loads such as chondrocytes whereas Sec24C alone is sufficient to cope at earlier stages of chondrocyte development. Alternatively, Sec24D might have a role in the transport of specialized cargo such as collagen irrespective of cargo load. Consistent with this second possibility, we found that collagen secretion is also stunted in notochord cells that produce and secrete moderate amounts of collagen (not shown).

A recent study in fruit fly revealed a novel mechanism of collagen VII concentration into transport vesicles [36]. The results show that the TANGO1 adaptor protein binds to collagen VII and ER membrane and facilitates slow packaging into the COPII vesicular carrier, which expands to accommodate the large cargo. This mechanism that allows for a dynamic, cargo-specific, vesicle formation could enable transport of proteins that do not contain transmembrane domains and have large sizes and/or inflexible structures such as collagen fibrils. It would be interesting to test whether Sec24D preferentially interacts with specialized adaptor proteins for collagen cargo such as TANGO1.

### The Deficits in ECM Deposition Affect Development of Chondrocytes

It has been previously shown that chondrocyte differentiation is regulated by adhesive interactions between cells and cross-talk between cells and extracellular matrix [20]. The ECM-cell interactions direct remodeling of adherens junctions, rearrangement of actin cytoskeleton and cell intercalations to form mature stacks of chondrocytes. Consistent with this scenario, histological analyses indicate that these processes fail in *bulldog* mutants. Moreover, the ECM secretory defects coincide with upregulation of genes in the collagen synthesis pathway such as the transcription factor Sox9a and its downstream target Col2α1. The compensatory overexpression of collagen could further aggravate the situation leading to severe protein backlog. Indeed, we observed accumulation of electron dense material in the ER of *bulldog* mutant chondrocytes suggesting that they fail to cope with the extra protein load. Excess protein might activate the ER stress response, which is defined as an imbalance between cellular demand for ER functions and actual ER capacity [19]. Cells under ER stress respond with induction of generic molecular chaperones like Bip and Sil1 and substrate-specific chaperones such as Hsp47, which is collagen-specific. Our results show elevated expression of *bip, sil1* and *hsp47* that is consistent with activation of stress response mechanisms. This condition has been also observed in various pathological states known to affect collagen homeostasis including progression of fibrosis, rheumatoid arthritis, and systemic sclerosis [37], [38]. Moreover, mutations in the human SIL1 protein cause the Marinesco-Sjögren Syndrome (MSS), which is characterized by mental retardation, ataxia, cataracts, myopathy and skeletal deformities including short stature, a set of symptoms similar to mutations in the COPII component SEC23A [5], suggesting that at least some of the observed abnormalities are due to activation of ER stress response mechanisms.

### Mutations in Various COPII Proteins Display Distinct Ultrastructural Anomalies

The ER was shown to contain functionally and structurally defined sub-domains to meet requirements of individual cell types for secretory protein biogenesis, lipid synthesis and calcium signaling. Examples of specialized sub-compartments include smooth ER (SER), rough ER (RER), nuclear envelope, and ER exits sites on RER [39], [40]. EM analyses showed interesting structural organization of SER [41], but less is known about RER spatial organization. The *bulldog* mutation revealed an additional level of RER complexity. By examining electron micrographs, we found that Sec24D-deficient chondrocytes maintain both normal and distended RER raising the possibility that Sec24D is responsible for trafficking of proteins synthesized in cargo-specific RER sub-domains. Because type II collagen is not secreted by *bulldog* chondrocytes, it is likely that RER areas with normal appearance is not involved in collagen biogenesis (Figure 3F). New lines of investigation may help us understand the precise mechanisms of RER sub-compartmentalization.

Similar paradigms of broad expression pattern and relatively tissue-specific loss-of-function phenotypes have been reported for other genes important for collagen secretion [42]. For example, mice with inactivation of perlecan, an ECM protein required for collagen fibril assembly, fail to organize cartilage growth plates after the mesenchymal condensation stage and the phenotype progressively worsens as development progresses. At the molecular level, these mice upregulate transcription of *collagen2α1* and *matrilin* and display sparse and disorganized ECM fibrils, distended RER and increased number of vesicles [43]. Similar phenotypes have been reported for mutations affecting collagen expression and processing. We also found that mutations in the Sec24D partner Sec23A have similar defects [6]. Thus, it appears that regardless of the mechanism leading to ECM depletion. (i.e., by failure to synthesize [44], [45], process [46], transport (Sec24D/*bulldog*, Sec23A), or assemble Collagen2α1 [47]), the cartilage phenotype is consistent on the molecular, cellular and developmental levels.

### Human Disorders Related to Protein Trafficking

Recently, human disorders have been linked to several components of the COPII complex. CMRD (Chylomicron Retention Disorder), which disrupts the function of SARA2, is characterized by fat malabsorption and failure to thrive in infancy, with a characteristic absence of chylomicrons in the blood stream [32]. Conversely, lesions in the SEC23A protein, which directly binds to SARA2, cause cataracts and skeletal malformations in CLSD (cranio-lenticulo-sutural dysplasia) patients [5]. In parallel, we have shown that the zebrafish mutation *crusher/sec23a* perturbs cartilage development causing a phenotypically similar craniofacial dysmorphology as observed in CLSD patients [6]. Finally, recent studies in patients with congenital dyserythropoietic anemia type II (CDAII) uncovered mutations in the *SEC23B* gene revealing its essential role in erythrocyte lineage development. However, the authors did not find erythrocyte defects in Sec23A-deficient zebrafish and concluded that SEC23A and SEC23B, although highly similar paralogs, play non-redundant roles in erythrocyte maturation [48]. These results further support the idea that different COPII components have highly specialized roles in organ development and homeostasis.

In conclusion, our findings bring new developmental perspective on the role of Sec24D that previous *in vitro* studies deemed dispensable for cargo transport [11]. Studies of model proteins will continue to provide important insights to biochemical aspects of protein transport. However, genetic approaches, which interrogate the entire organism in an unbiased way, will be instrumental to understand principles of cargo trafficking *in vivo*.

## Materials and Methods

All experiments were conducted in accordance with the guidelines established by the IACUC Committee at the Vanderbilt University and the University of Freiburg.

### Zebrafish Maintenance and Breeding

Fish were raised and maintained as previously described [49]. *bulldog* mutations were induced and recovered during the large-scale chemical mutagenesis screen carried out in Boston [13]. The *bulldog* alleles (*bul^m757^*, *bul^m606^*, *bul^m494^*, *bul^m421^*) were kept in the AB* genetic background for phenotypic analysis and crossed to the HK and IN lines for genetic mapping. The standard staging of zebrafish embryos is used and determined in hpf (hours post fertilization) or dpf (days post fertilization) at 28°C [50]. For most of the experiments we show results obtained in the *bul^m606^* line or as indicated; all groups of experiments were duplicated in the four alleles.

### Genetic Mapping and Cloning

The *bulldog* locus was mapped in a F2 intercross using bulked segregate analysis. DNA samples were PCR-genotyped with SSLP markers evenly spaced across the zebrafish genome. The mapped *bulldog* mutations were confirmed by sequencing genomic DNA from three homozygous wild type F2 animals, six heterozygous F2 animals, six homozygous mutant F2 animals, and six different pools of cDNAs from either homozygous/heterozygous F2 animals, or homozygous mutant F2 animals. Primers used for sequencing are listed in Table S1. The sequence of Sec24d and Sec24c was compiled using zebrafish ESTs and trace repository data (NCBI). The full-length clones were PCR amplified, sequenced and submitted to the GenBank Database: Sec24c BankIt1322644 GU90849; Sec24d BankIt1324830 GU944484.

### Morpholino Oligonucleotides

Antisense morpholino oligonucleotides (MOs) (Gene Tools) were designed to target the *sec24d* 5′UTR (sec24d-MO), the *sec24c* 5′UTR (*sec24c* UTR-MO), or the *sec24c* translation start site (*sec24c* ATG-MO); sequences are included in Table S1. MO concentrations were determined spectrophotometrically and 1 nl was injected into 1–2 cell stage embryos at increasing doses (0.5 ng–10 ng) to determine optimal concentrations. Working doses for each MO are indicated in the figure legends. Specificity and effectiveness were tested for each morpholino.

### Cartilage Proteoglycan Staining

Alcian Blue staining was done as previously described [49]. Embryos at 4 dpf were fixed in 4% phosphate-buffered paraformaldehyde (PFA), bleached in 10% H_2_O_2_ and 1 M KOH, and stained overnight in 0.1% Alcian Blue solution.

### Histology

Embryos were fixed in 4% PFA, dehydrated to 95% ethanol, embedded in JB-4 resin (Polysciences), sectioned at 5 µm thickness using a Leica RM2265 microtome and stained with the metachromatic dye toluidine blue.

### *In situ* Hybridization

Whole-mount *in situ* hybridization with probes recognizing *col2a1*, *sox9a* and *hsp47* was performed as previously described [6]. The *sec24d* probe was made by cloning 386 nucleotides from the 3′UTR of *sec24d* cDNA into the pBS SKII (+) (Stratagene) vector with primers 5′GCTTCGTCCACCGCGAGATCC3′ and 5′GGTGTGATGATTTCATCCCTGAAGTC3′. The *sec24c* probe was made by cloning 460 nucleotides from the 3′UTR of *sec24c* cDNA into the pGEM®-T Easy vector with primers 5′GATTCGTCAGCTCCTGAGCTGAG3′ and 5′CACAGAACACCCAGTACAATCAAC3′.

### Transmission Electron Microscopy (TEM)

For TEM, embryos were fixed in 2.0% glutaraldehyde and 1.0% paraformaldehyde in PBS. Specimens were post fixed with 1.0% osmium tetroxide, stained with 1.0% uranyl acetate and analyzed in the Vanderbilt Electron Microscopy Core Facility.

### Immunohistochemistry, Peanut, and Wheat Germ Agglutinin Staining

For whole mount staining, embryos were fixed in 4% PFA, 0.15 mM CaCl_2_, 4% sucrose in 0.1 M phosphate buffer (pH 7.3) and treated with Proteinase K at 4°C overnight. Immunofluorescence experiments were performed as we previously described [49] with 1∶100 diluted primary antibodies against collagen type II (Polysciences) and Alexa Fluor 555 fluorescently conjugated secondary antibody (1∶400; all Alexa-conjugates were from Molecular Probes). For immunohistochemistry on sections, embryos were fixed as above, embedded in 1.5% agarose in 5% sucrose, and stored in 30% sucrose solution at 4°C overnight. Agarose blocks were mounted with O.C.T. (Sakura Finetechnical Co.). Twenty micrometer sections were cut using a Leica CM 3050 cryostat at −20°C and transferred onto *Superfrost* slides (Fisher). Sections were washed in PBS, blocked in 2 mg/ml BSA, 2% goat serum, 2% DMSO in PBS and incubated with primary antibodies (1∶250 dilution) against Fibronectin, Pan-cadherin, β-catenin (Sigma) and β1-integrin (Chemicon International) at 4°C overnight. Alexa Fluor 555 IgG conjugate was applied as secondary antibody (1∶500). In chondrocytes, the N-cadherin is the predominant protein recognized by polyclonal Pan-cadherin antibodies. Peanut Agglutinin (PNA) staining was conducted on cryo-sections with PNA-Alexa Fluor 488 conjugate (1∶250). PNA recognizes β-D-gal-NAc-D-galactose, a terminal carbohydrate moiety on cell surface glycoproteins or proteoglycans providing a global assessment of glycosylated proteins at mesenchymal condensation stage [25]. Wheat Germ Agglutinin (WGA) staining was conducted with WGA-Alexa Fluor 488 conjugate (1∶250). The WGA lectin binds to N-acetylglucosamine and N-acetylneuraminic acid residues of membrane and matrix glycoproteins. TOPRO-3 (Molecular Probes) was used for nuclear counterstaining. Confocal images were taken with a Zeiss LSM510 Meta laser-scanning microscope (Vanderbilt Cell Imaging Shared Resource).

### Quantitative PCR Analysis

Total RNA was extracted from approximately 100 embryos at different embryonic time points using the TRIzol reagent (Sigma). Three micrograms of total RNA were reverse transcribed to cDNA using M-MLV reverse transcriptase (Promega). Each PCR reaction was performed with 1 µl of cDNA using iQ™ SYBR Green Supermix (Bio-Rad) and 5 µM of each primer. Primer sets used are listed in Table S1. Three independent experiments in triplicates were performed using β*-*actin as internal control. Thermal cycling was carried out in an iQ 5 (Bio-Rad) and relative expressions were calculated following previously described methods [51].

### Cell Measurements and Counts

Embryos from *bulldog* heterozygous crosses were injected with synthetic RNA (200 pg) encoding membrane-GFP [the Ras membrane-localization sequence (CAAX) fused to the C terminus of GFP] at the one-cell stage to mark cell membranes [52]. Confocal Z-stacks of chondrocytes from live wild-type and *bulldog* embryos at 3 dpf were imaged and collected for analysis using the ImageJ software (NIH). Total cell number and their distribution in the ceratohyal cartilage were counted at 5 dpf in six wild-type and six *bulldog* embryos that were Alcian blue stained. Progress of intercalations was assessed by counting the number of cells contacting both lateral boundaries of ceratohyal (one cell), and by taking into account how many cells were spanning the width of the ceratohyal in one straight line (one, two, three or four cells). Measurements of the longest and shortest axes of the cells were exported to Excel (Microsoft) in order to calculate average length, width and length-to-width ratios. Data represent results of three different wild-type and *bulldog* embryos.

### Statistical Analysis

Data in bars represent average ± s.d. Statistical analyses were performed using unpaired two-tailed Student's *t*-test (GraphPad Software) and *p* values <0.001 were considered as significant.

## Supporting Information

Figure S1 Alignment of Predicted Protein Sequences of Sec24D. Sequences from Homo sapiens, Mus musculus, Takifugu rubripes and Danio rerio using the ClustalW program (SDSC Biology WorkBench). Identical residues (*), conserved (:), and semi conserved (.) substitutions are marked. Domain structures are color-coded as follows: zinc finger domain in grey, trunk domain in dark red, β-barrel domain in yellow, α-helical domain in turquoise and gelsolin-like domain in green. The accession numbers of the protein sequences are: NP_055637(H. sapiens), NP_081411(M. musculus), and SINFRUG00000143757 (Ensembl) (T. rubripes). The zebrafish sequences submitted to the Genbank Database: Sec24c BankIt1322644 GU90849; Sec24d BankIt1324830 GU944484.
(0.09 MB PDF)

Figure S2 Sense control for the expression of sec24d in 3 dpf embryo by in situ hybridization shows no staining. Adherence Junctions Persist in bulldog Mutant Chondrocytes. (A–D) Pan-cadherin antibody predominantly labels N-cadherin in 3 dpf chondrocytes (green). Cadherin staining disappear as wild-type chondrocytes begin to lose cell-cell contacts (arrow in A,B). In contrast, bulldog chondrocytes (C,D) express uniform levels of Cadherin in all cells that is concentrated at cell-cell contact points (arrowheads). Nuclei are stained blue with TOPRO-3. Scale bars are 5 µM.
(0.35 MB TIF)

Figure S3 Cartilage Matrix Proteins are not Trafficked in bulldog Chondrocytes. Loss of Sec24D blocks ECM protein secretion in mature chondrocytes. (A–F) Overview of the lower jaw at 59 hpf labeled for Col2α1 in wild-type and bulldog embryos. The head is compact in wild types and laid out in more cuboidal shape, thus appearing smaller in the images (A,C,E), as compared to bulldog jaws that are wider and more flat along the Z axis (B,D,F). Type II collagen (A,B) is not trafficked in bulldog chondrocytes (B) at 59 hpf. Boxed areas are enlarged as insets. Wheat Germ Agglutinin (WGA)-labeled glycoproteins (C,D) are primarily localized to the extracellular space in the epithelial membranes of wild-type and bulldog embryos, but are weakly expressed in bulldog chondrocytes. The corresponding superimposed images are shown in E, F. Nuclei are stained blue with TOPRO-3. The insets in E,F are higher magnifications showing that the two labels mark distinct compartments, with WGA likely staining the Golgi complexes and the Col2α1 antibody the ER. (G–L) Co-staining of Col2α1 and WGA at 4 dpf in the first pharyngeal arch cartilages shows colocalization in the extracellular space in wild types (G–K), and accumulation in large vesicular structures in bullog mutants (H–L). (M–R) Matrilin, an extracellular matrix protein, is localized in the extracellular space of the basihyal cartilage and in tissues surrounding individual cartilage elements at 5 dpf in wild types (M), but it accumulates within cells in bulldog embryos (N). Boxed areas are enlarged. Counterstaining with WGA is shown in O and P. The large, round stained dots in wild-type and bulldog samples are the mucin producing cells of the digestive system present in both whole mount preparations (arrowheads). Abbreviations: m: Meckel's cartilage; bh - basihyal cartilages; ch - ceratohyal cartilages. Scale bars are 20 µM. Methods: Immunofluorescence experiments were performed with 1∶250 diluted primary antibodies against Matrilin (generous gift of Dr. E. Kremmer). Anti-rat Alexa Fluor 555 (Invitrogen) was applied as fluorescently-conjugated secondary antibody (1∶400).
(3.67 MB TIF)

Figure S4 Sec24c knockdown strategy. (A,B) Schematic representation of the knockdown strategy for sec24c and the location of the translation blocking morpholinos: UTR-MO (A), ATG-MO (B). (C) Schematic drawing of the construct containing 238 bp of sec24c mRNA fused in frame to three HA tags in the pCS2+ vector that was used to test the effectiveness of translation inhibition by the morpholinos. (D,E) Injection of 100 pg of the HA tagged construct into 1-cell stage embryos shows expression as visualized by HA tag recognizing antibody in red (D). Injection of 100 pg of the HA tagged construct in combination with 2.0 ng of MO effectively blocked translation of the reporter construct (E).
(0.19 MB TIF)

Table S1 (0.05 MB PDF)
