# Supplementary material for: Sec24D-Dependent Transport of Extracellular Matrix Proteins Is Required for Zebrafish Skeletal Morphogenesis
Source: PLoS One. 2010 Apr 28;5(4):e10367. doi: 10.1371/journal.pone.0010367 (PMC2860987; doi:10.1371/journal.pone.0010367)
Supplement: Figure S1 — Alignment of Predicted Protein Sequences of Sec24D. Sequences from Homo sapiens, Mus musculus, Takifugu rubripes and Danio rerio using the ClustalW program (SDSC Biology WorkBench). Identical residues (*), conserved (:), and semi conserved (.) substitutions are marked. Domain structures are color-coded as follows: zinc finger domain in grey, trunk domain in dark red, β-barrel domain in yellow, α-helical domain in turquoise and gelsolin-like domain in green. The accession numbers of the protein sequences are: NP_055637(H. sapiens), NP_081411(M. musculus), and SINFRUG00000143757 (Ensembl) (T. rubripes). The zebrafish sequences submitted to the Genbank Database: Sec24c BankIt1322644 GU90849; Sec24d BankIt1324830 GU944484. (0.09 MB PDF) [file pone.0010367.s001.pdf]

The Sec24D primary structure is highly conserved during evolution

```
H. sapiens     MSQQGYVATPPYSQPQPGIG---------LSPPHYGHYGDPS--HTASPTGMMKPAGPLG   49
M. musculus    MSQQGYVATPPYSQSQPGMG---------ISPPHYGHYGDPS--HASSPPGVMKPLGPS-   48
T. rubripes    MSQQGYVAAPPYSQAQPRMGGYQGGFGPGPAQPMNGHYGGPPQAFTAPPTGMMKPP----   56
D. rerio       MSQQGYVATPPYSQAQAGMGGYAAGFGNPPPQSHYGAYGSPTQGYSAPPTGVLKPP----   56
               ********:*****.*. :*          . .  * **.*.  .::.*.*::**

H. sapiens     ATATRGMLPPGPPPPGPHQFGQNGAHATGHPPQRFPGPPPVNNVASSHAPYQPSAQSSYP  109
M. musculus    -AAPSGMLPPGPLPPGPPQFGPNGAHTPGHPPQRFPGPPPVNSVAPSYASGQTLPQSSYP  107
T. rubripes    -VSS-GAMPP---PP--SQFSPN-MQHNG--PQSYP------AASAAYGQPP---YNSMA   97
D. rerio       -ASSPSGMPP---PPVSSQYGTN-IQQNGAHPHSFPPPVSSSPSMSPYGQPPPTAFHSMA  111
                .:. . :**   **   *:. *  :  *  *: :*         ..:.       * .

H. sapiens     GPISTSSVTQLGSQLSAMQINSYGSGMAPPSQGPPGPLSATSLQTPPRPPQPSILQPGSQ  169
M. musculus    GPGSTSSVTQLGSQFSSMQINSYGVGATPQSQGPPGPQSAGAFQGPPQPAQPSILQPGHQ  167
T. rubripes    S-QAPPPTQHLTNQMSAMNLGSYVMGQP---QMPPGPQSP--QMSPPQSPSASMPMGGPP  151
D. rerio       --QAPPPTQQLTNQMSAMNIAGYGQRPV---QSPSNSAPA--HFQTSPPP---PVMGHPP  161
                  :.... :* .*:*:*:: .*        * *... ..     ..  ..

H. sapiens     VLPPPPTTLNGPGASPLPLPMYRPDGLSGPPPPNAQYQPPPLPGQTLGAGYPPQQAN---  226
M. musculus    VPPPPPTALNGPGASPMSPPTHRQDGLPGPAPLNAQYQPPPPPGQTLGPGYPPQQATN--  225
T. rubripes    MAGPPMAGPPMAG-PPMAGMG-RP--FPGPSPPGPGGFPQPSPGTAVPPGYPQQPGQ---  204
D. rerio       LSPPGQQPSPLGSPPPMATMGSMP--APPMGMSGPPGPVVHQP-PHGPQGYPQQPGSPIA  218
               :  *        . .*:.         .     ..       *      *** * .

H. sapiens     --SGPQMAGAQLS------YPGGFPGGPAQMAGP-PQPQKKLDPDSIPSPIQVIENDRAS  277
M. musculus    --YGPQMGGAQMS------YPGGFPGGPAQMAGPAPQLQRKLDPDSIPSPIQVIENDRAT  277
T. rubripes    --FGGLIAGPQQG------MPGAFPGAPGGLAGP---PQKKLDPDSIPSTNRCVWQTQXD  253
D. rerio       GPYGAQMAGPQMAGPQTGAFPGGFAGGPAQMAGP---PQKKLDPDSIPSTTQMIADDQAK  275
                  *  :.*.* .       **.*.*.*. :***    *:*********. : : : :

H. sapiens     RG-------GQVYATNTRGQIPPLVTTDCMIQDQGNASPRFIRCTTYCFPCTSDMAKQAQ  330
M. musculus    RG-------GQVYTTNTRGQVPPLVTTECVIQDQGHSSPRYIRCTTYCFPCTSDMAKQAQ  330
T. rubripes    RGRPDQNTGGRSKTTNIRGQVPPLVTTDFTVQDQGNASPRYMRCTAYSLPTTADLAKQCQ  313
D. rerio       RG-------GQVYVTNIRGQVPPLVTTNFTVQDQGNASPRFMRCTTYSFPCTADLAKQCK  328
               **       *:  .** ***:******:  :****::***::***:*.:* *:*:***.:

H. sapiens     IPLAAVIKPFATIPSNESPLYLVNHGESGPVRCNRCKAYMCPFMQFIEGGRRYQCGFCNC  390
M. musculus    IPLAAVIKPFADIPPNETPLYLVNHGESGPVRCNRCKAYMCPFMQFIEGGRRYQCGFCSC  390
T. rubripes    VPLATIITPLAALPKNEAPLYLVNHGETGPIRCNRCKAYMCPYMQFTDGGRRYQCSFCNC  373
D. rerio       VPLAAIIKPFATVPKNETPLYVVNHGETGPIRCNRCKAYMCPYMQFIDGGRRFQCSFCSC  388
               :***::*.*:* :* **:***:*****:**:***********:*** :****:**.**.*

H. sapiens     VNDVPPFYFQHLDHIGRRLDHYEKPELSLGSYEYVATLDYCRKSKPPNPPAFIFMIDVSY  450
M. musculus    VNEVPPFYFQHLDHIGRRLDHYEKPELSLGSYEYVATLDYCRKNKPPSPPAFIFMIDVSY  450
T. rubripes    VNEVPVFYFQHLDHMGRRVDFYERPELSLGSYEFVATLDYCKNNKPPNPPAYIFMIDVSY  433
D. rerio       VNEVPVHYFQHLDHMGRRMDLYERPELSLGSYEFAATLDYCKNNKPPNPPAYIFMIDVSY  448
               **:** .*******:***:* **:*********:.******::.***.***:********

H. sapiens     SNIKNGLVKLICEELKTMLEKIPKEEQEETSAIRVGFITYNKVLHFFNVKSNLAQPQMMV  510
M. musculus    SNIKNGLVKLICEELKTALKRLPKEEHEETSAIRVGFITYNKVLHFFNVKSNLAQPQMMV  510
T. rubripes    NNVKSGLVKLICEELKTLLQNLPREDGADASAIKVGFVTYNKILHFYNVKSALAQPQMMV  493
D. rerio       NNIKSGLVRLICEELKTLLDRLPKEEGAESSSIKVGFVTYNKILHFYNVKSALAQPQMMV  508
               .*:*.***:******** *..:*:*:  ::*:*:***:****:***:**** ********

H. sapiens     VTDVGEVFVPLLDGFLVNYQESQSVIHNLLDQIPDMFADSNENETVFASVIQAGMEALKA  570
M. musculus    VTDVGEVFVPLLDGFLVNYEESQSVIHNLLDQIPEMFADSNENETVFAPVIQAGMEALKA  570
T. rubripes    VSDTAEMFVPLQDGFLVSYQESRAVISNLLDQIPDMFADTGESETVFAPVIQAGIEAFKA  553
D. rerio       VSDTAEMFVPLLDGFLVNFQESRAVINNLLDQIPDMFADTNESETVFAPVVQAGLEALKA  568
               *:*..*:**** *****.::**::** *******:****:.*.*****.*:***:**:**

H. sapiens     ADCPGKLFIFHSSLPTAEAPGKLKNRDDKKLVNTDKEKILFQPQTNVYDSLAKDCVAHGC  630
M. musculus    AECPGKLFIFHSSLPTAEAPGKLKNRDDKKLVNTDKEKILFQPQTAVYESLAKDCVANSC  630
T. rubripes    AQCSGKLFIFHSSMPTAEAPGKLKNRDDKKLVGTDKEKTLFQPQKGVYEQLSKECVAQGC  613
D. rerio       AECSGKLFIFHSSIPTAEAPGKLKNRDDKKLVGTEKEKTLFQPQRGVYEQLTKDCVAQGC  628
               *:*.*********:******************.*:*** *****  **:.*:*:***:.*

H. sapiens     SVTLFLFPSQYVDVASLGLVPQLTGGTLYKYNNFQMHLDRQQFLNDLRNDIEKKIGFDAI  690
M. musculus    SVTLFLFPSQFVDVASLGLVPLLTGGTLYKYNVFQIHSDSQRFLTDLRNDIEKKIGFDAI  690
T. rubripes    CVDLFLFPSQYVDVSTMGDVPARTGGSVYKYSNFQVDVDGEHFLRDLRKDVQKRVGFDAI  673
```

```
D. rerio         CVDLFLFPNQYVDIATMGDVPSHTGGSIYKYSNFQVEVNGQQFLSDLSRDVEKSIGFDAI   687
                 .* *****.*:**::::* **  ***::***. **:. : ::** ** .*::* :*****

H. sapiens       MRVRTSTGFRATDFFGGILMNNTTDVEMAAIDCDKAVTVEFKHDDKLSEDSGALIQCAVL   750
M. musculus      MRVRTSTGFRATDFFGGIFMNNTTDVEMAAIDCDKAVTVEFKHDDKLSEDVGALIQCAVL   750
T. rubripes      MRVRTSTGFRATDFFGAIHMNNTTDVEMAAVDCDKAVTVELKHDDALNEEAGALLQCALL   733
D. rerio         MRVRTGTGFRATDFFGAVYMNNTTDVEMAAVDCDKAVTVEFKHDDTLSEESGAVMQCALL   748
                 *****.**********.: ***********:*********:**** *.*: **::***:*

H. sapiens       YTTISGQRRLRIHNLGLNCSSQLADLYKSCETDALINFFAKSAFKAVLHQPLKVIREILV   810
M. musculus      YTTISGQRRLRIHNLALNCSTQLADLYKSCETDALINFFAKSAFKAVLNQPLKAIREILV   810
T. rubripes      YTTIGGQRRLRVHNLSLNCSSQLSELYKSCETDALINFFAKSAYRAILNQPLKSVREILV   793
D. rerio         YTTIGGQRRLRIHNLSLNCSSQLSELYKSCETDALINFFAKSAYRAMLNQPMKTVREILV   808
                 ****.******:***.****:**::******************::*:*:**:* :*****

H. sapiens       NQTAHMLACYRKNCASPSAASQLILPDSMKVLPVYMNCLLKNCVLLSRPEISTDERAYQR   870
M. musculus      NQTAHMLACYRKHCASPSAASQLILPDSMKVLPVYMNSLLKNCVLLSRSEISPDERAYQR   870
T. rubripes      NQTAHMLACYRKNCASPSAASQLILPDAMKVFPVYMNSLMKTGPLVGSTELSTDDRAHQR   853
D. rerio         NQTAHMLACYRKNCASPSAVSQLILPDAMKVFPVYMNSLLKTPALVSSTELSTDDRAFHR   868
                 ************:******.*******:***:*****.*:*.  *:. .*:*.*:**.:*

H. sapiens       QLVMTMGVADSQLFFYPQLLPIHTLDVKSTMLPAAVRCSESRLSEEGIFLLANGLHMFLW   930
M. musculus      QLVMTMGVADSQLFFYPLLLPIHTLDVKSAALPPAVRCSESRLSEEGIFLLANGLNMFLW   930
T. rubripes      LAVMAMGVEETQLLLYPRLIPLHNMDVGAEAPPTPLRCSEERLADAGVFLLENGHAMFLW   913
D. rerio         FMVNAMGVEETQVLLYPRLIPLHTMDVSSESLPAAVRCSEERLNESGMFLLENGLSMFLW   928
                   * :*** ::*:::** *:*:*.:** :   *..:****.** : *:*** ** ****

H. sapiens       LGVSSPPELIQGIFNVPSFAHINTDMTLLPEVGNPYSQQLRMIMGIIQQKRPYSMKLTIV   988
M. musculus      FGVGSPPELIQGIFNVPSFAHINTDMASLPEVGSPHSQQLRMIMNNIQQKKPYSMKLIVV   990
T. rubripes      LGQASPPDLIQNLFNVPSLAHLQAHMSVLPVLDNPLSKKVRGVIGDLLEKRPGSMKLQIV   971
D. rerio         LGQACSPDLIQNLFNVPSFGHLPTEGTSLPVLDNAHSRKLHSIISRISQQRAASMKLLIV   988
                 :* ...*:***.:*****:.*: :. : ** :... *:::: ::. : :::. **** :*

H. sapiens       KQREQPEMVFRQFLVEDKGLYGGSSYVDFLCCVHKEICQLLN   1032
M. musculus      KQREQREMAFRQFLVEDKGLYGGSSYVDFLCCVHKEICQLLN   1032
T. rubripes      RQKDKPEMLFRQFLVEDKGLHGGASYMDFLCYIHREVRQLL-   1014
D. rerio         KQKDRAEMSFRQLLVEDKGLHGGASYMDFLCFVHREIRQLLT   1030
                 :*::: ** ***:*******:**:**:**** :*:*: ***
```

Sarmah et al., Figure S1
