# Supplementary material for: Sec24D-Dependent Transport of Extracellular Matrix Proteins Is Required for Zebrafish Skeletal Morphogenesis
Source: PLoS One. 2010 Apr 28;5(4):e10367. doi: 10.1371/journal.pone.0010367 (PMC2860987; doi:10.1371/journal.pone.0010367)
Supplement: Table S1 — (0.05 MB PDF) [file pone.0010367.s005.pdf]

Table S1.

Sequences of primers and morpholino oligonucleotides used in this study

Oligonucleotide	Sequence
F1	5′CAGTAAGATGAGTCAGCAAGG3′
F2	5′GGTCAAAGGCCTGTGCAGTCTCC3′
F3	5′CAAGGGAATGCCAGCCCGAGG3′
F4	5′GACGCTGCTTGACCGACTTCC3′
F5	5′CCCAATCAGTATGTGGATATCGC3′
F6	5′CGTGAGGGAAATCCTGGTCAACC3′
F7	5′CCTATGGCTAGGACAGGCCTG3′
R1	5′GGAGACATAGAAGGAGATGAAG3′
R2	5′CAGGTGACCAGAGGCGGCACCTG3′
R3	5′CGCATATTAGTCTGACAAGTCC3′
R4	5′GGAAGAGGTCGACACAACATCCC3′
R5	5′CGGCTGGTTGAGCATCGCCCG3′
R6	5′GACACATCCATAGTGTGCAGCGGG3′
R7	5′CTGTGAGAGGGTTAAGTGAGC3′
β-actin-F	5′-CCATGGATGAGGAAATCGCTGC-3′
β-actin-R	5′-GTCACACCATCACCAGAGTCC-3′
S3UTRF	5′GCTTCGTCCACCGCGAGATCC3′
S3UTRR	5′GGTGTGATGATTTCATCCCTGAAGTC3′
BipF	5′CAGGAAAGAGTAAAACAGCAACCG3′
BipR	5′CCGAAATTTTGCTCTCACTGCATC3′
Sil1F	5’CAGGAAAGAGTAAAACAGCAACCG3’
Sil1R	5’CCGAAATTTTGCTCTCACTGCATC3’
24cF	5′GATTCGTCAGCTCCTGAGCTGAG3′
24cR	5′CACAGAACACCCAGTACAATCAAC3′
3cutrf	5′GATTCGTCAGCTCCTGAGCTGAG3′
3cutrr	5′CACAGAACACCCAGTACAATCAAC3′

Morpholino oligonucleotides

Sec24d-MO	CTTACTGTTATACCTCTTTCATTCC
Sec24c-ATG-MO	GGTTGACATTCATTCTCAGTTCATC
Sec24c-UTR-MO	CCTAATCTCACAAACTGCTTGATTG
